# Pittsburgh Obstetrical Society

**Published:** 1890-04

**Authors:** 


### Society Reports.

### PITTSBURGH OBSTETRICAL SOCIETY.

Regular Meeting, November 7th, 1889.

R. Stansbury Sutton, M. D., President, in the chair.

Dr. Green: (Paper on Face Presentation.) See page 82.

Dr. Kearns: I never saw but one case of face presentation. It is rarely encountered. I have had cases which were face presentations, but not worthy of mention, inasmuch as they were premature fœtuses, and not attended with any difficulty whatever. The one case I had, thirty years ago, I will never forget. I had had but little experience prior to that time. The labor was protracted and very severe. Delivery was spontaneous. When I recognized the presentation it was far advanced. I cannot recollect whether the child was dead or living; my memory is at fault. But I know the patient recovered, although it required a vast amount of patience on the part of the attendants, the case being protracted. I am sorry the doctor did not go more fully into the treatment of these cases. His classification of three forms is perhaps practically correct, posterior, right and left. As to the cause of this mal-presentation, might it not be automatic? Might not the motions of the child produce it? Might not the fœtus take a somersault and turn around and never be restored? With regard to treatment of cases, their delivery should be left to nature, I think.

Dr. Blume: I was much pleased with Dr. Green's paper. As he says, face presentation has often been the subject of discussion among obstetricians, and yet there is still a diversity of opinion as to its etiology and treatment. The doctor gave some causes which may produce this presentation. Winckel states that there are thirty-three theories on the etiology of face presentation. I do not intend to dwell on these, I wish to make a few remarks on the treatment. Since Boer, in 1791, first clearly described the mechanism of face presentation and showed that the chin always rotates to the front, no matter in what diameter the face presents at the pelvic inlet, an expectant treatment has more generally been recommended. To-day it is the opinion of leading authorities that an expectant management should be considered as a fundamental rule in all those cases in which no contra-indication exists at the commencement of labor and no complication develops during the process of labor.

Such a contra-indication is a contracted pelvis. Its importance in the etiology of face presentation is well known, for we meet with it in about one-third of all cases. Another important contra-indication is placenta prævia. The third factor, which often may require interference, is feebleness of the uterine action, which may develop during the progress of labor, and finally, anomalies of the mechanism will also require assistance,—among them cases of mento-posterior positions in which anterior rotation does not occur. Let us consider now a case with a contracted pelvis, where the true conjugate is somewhat less than four inches. It is the opinion of most authorities that the delivery should not be left to the powers of nature. The best method to deliver the child and protect the mother in such cases is version. If this fails, it may be possible to change the extension of the head and produce flexion. If this is a failure and the chin does not rotate to the front, it might be permissible for a skilled man to apply forceps and try to perform rotation, but very carefully. The blades may be applied like Scanzoni recommends and may be taken off and re-applied if necessary. If this also should fail and the condition of the mother demands prompt delivery, craniotomy remains as the only safe procedure.

Now, in cases of normal face presentation, where the head and pelvis are normal, in such cases rotation usually will take place and no interference is necessary as long as the mother and child are in good, healthy condition. If rotation should not occur we have several ways to imitate and assist nature. In cases of mento-posterior presentation, for instance, rotation may not take place, because, as Penrose recently described, the chin cannot descend far enough to meet with a sufficient resistance, and this is certainly true. He recommends a simple procedure, which deserves our attention. He applied in such a delayed case a blade of the forceps on the posterior cheek so that it found re-sistance, and the chin swept around instantly. Another method is the application of the forceps and trying to bring the chin to the front in the manner described by Scanzoni, but, as Penrose terms it, coax it rather than compel it to rotate. A better method than this latter attempt to produce rotation is to produce flexion, and make out of a face presentation a normal vertex presentation. This can be done in three different ways. The old way, followed hundreds of years, is described by Dr. Green. The hand is introduced, the head lifted upward and the chin brought to the breast. If this fails, an attempt can be made to pull the occiput down, while the other hand presses firmly upon the head, through the abdominal walls. A better but more difficult method is described and recommended by Schatz. He changes the unnatural lordotic attitude of face presentations into the normal kyphotic attitude of vertex presentations by external manipulations.

This method requires great dexterity, is permissible only during the first stage of labor, and often impossible when the abdominal walls are very thick, so that it is difficult to make out the shoulders so that you have a hold on them. Schatz first showed that face presentation is not only a faulty position of the head, but a faulty position of the whole body.

The last method was described three years ago by Thorn, formerly assistant of Olshausen. The hand is introduced inside the vagina, the head lifted upward, the chin, the forehead and the occiput moved toward the breast. The external hand assists by pressing first the occiput downward, then the breast,—as you see here on the board—from left to right, and finally the breech from right to left, so that a completely normal vertex presentation is produced.

This method is said not to be difficult, it can be performed even after the waters are drained off. If these methods fail, podalic version is indicated, and should this prove impossible, the obstetrician having been called too late, nothing is left but craniotomy; it is the only safe way to deliver, and in such a case a conservative operation. It is not allowable under any condition to attempt to deliver with forceps when the chin is directed to the sacrum.

DR. DUFF: I would like to put myself on record. I don't believe in this conservative work in face presentation, as placed before us to-night. I think it the duty of the obstetrician to make his diagnosis early, and having made the diagnosis, rectify it by one of the methods suggested by Dr. Blume. You have not any right to sacrifice both mother and child by a faint hope that the head is going to pass through under these circumstances, where you have the opportunity of making a diagnosis early in the case. And, furthermore, I was sorry that Dr. Green did not go more extensively into consideration of the delivery of face presentation with chin posterior. The cases reported in our journals I do not believe. I believe it is an absolute impossibility for a child weighing 13½ pounds to pass through a pelvis, even if that pelvis is 6½ inches.

DR. BLUME: Dr. Green stated in his paper that external examination in face presentation fails, if I understood him aright. I must say that abdominal examination is often the only way to make out an exact presentation. The head is high up if you have a contracted pelvis, and it is sometimes impossible to diagnosticate exactly by the vagina those conditions which may be readily made out by abdominal palpation and auscultation.

DR. GREEN: I agree with Dr. Kearns in his remarks to a great extent. I think myself, and I believe the general impression is, where the pelvis is not contracted that all cases would terminate successfully without any surgical interference. It may have been my misfortune to meet with a few more face presentations than some others, yet I have not seen nearly so many as some physicians who attend a much less number of cases of confinement than do I. I have never seen a delivery where rotation of the head did not occur, though I have no reason to disbelieve such men as have testified differently. I think certainly they know what they saw. I have only one remark to make in regard to what Dr. Blume has said as to deformed pelvis. So far as I have looked up the matter, I think his report of causes is true. A deformity of the pelvis, whilst it may produce a face presentation, is no more likely to produce a face presentation than any other position of the head, and whilst it may produce a larger number of cases than some of the other causes assigned, I would state that so far as I have read the matter up, the authors do not so state it. One further remark I wish to make in regard to the causes spoken of by Dr. Kearns, that is, the motion of the child itself. Now that is given by some author, I cannot tell you by whom. Dr. Blume speaks of 33 theories (Winckel). There are more than that number. As regards version, in the cases that I saw, and so far as I have looked the matter up, version after the rupture of the membrane by external means, is not possible; it has failed entirely in the hands of the finest obsteticians. Besides, version of the child is one of the most hazardous operatians to the mother. More women die after version than after any other surgical procedure unless it be after craniotomy. They can be lacerated to a great extent by the use of instruments, or by manipulation in trying to place the head in position. During anæsthesia they will endure instruments for hours, and they will endure the powerful manipulations of three or four men working for hours, and will recover, yet after version the mortality is greater.

DR. DUFF: Do you think that statistics stating the number of deaths from version during the past fifteen years would be reliable to-day? One of the great causes of mortality from version in the old statistics is the fact that we did not use antisepties.

DR. GREEN: I am glad you spoke of that. In looking this matter up I found one writer who stated that with all the present antiseptic facilities the mortality was greater.

DR. BLUME: I only want to defend version. The obstetrician more and more recognizes the value of version in some cases, but these cases must be suitable ones; you must not have a half-dead woman to practice on.

DR. SUTTON: I want to take up the point Dr. Duff started. I say the statistics in obstetrics, beginning ten years ago and going back beyond, are worth nothing. Beginning ten years ago, and coming this way, in the days of cleanliness, antiseptics, and chloroform, and the whole thing is changed. Version is as safe an operation ordinarily as putting your hand in the uterus and taking out the placenta.

DR. DUFF: In substantiation of that we have the statistics before us to-day, that in certain hospitals where the death rate was as high as 17 per cent. ten years ago, it is now less than one-half of one per cent.

DR. SUTTON: Before closing the meeting I want to invite your attention to two specimens of large tumors.

This one is a fibroid, with the fundus of the uterus attached, the mass weighs 9 lbs, and was removed two days ago at my private hospital by supra-vaginal hysterectomy. The second one is a multilocular ovarian cyst. The mass contains more cysts than I have ever before seen in one specimen. It was removed a few days ago. My results in ovariotomy now stands 41 cases without a death, and 47 cases with one death.

